# Community engagement in eye care: working with and for communities

**Published:** 2022-09-20

**Authors:** Kriti Shukla

**Affiliations:** Research Associate: Centre for Health Outcome Research and Economics, Indian Institute of Public Health, Hyderabad, India.

**Figure:**
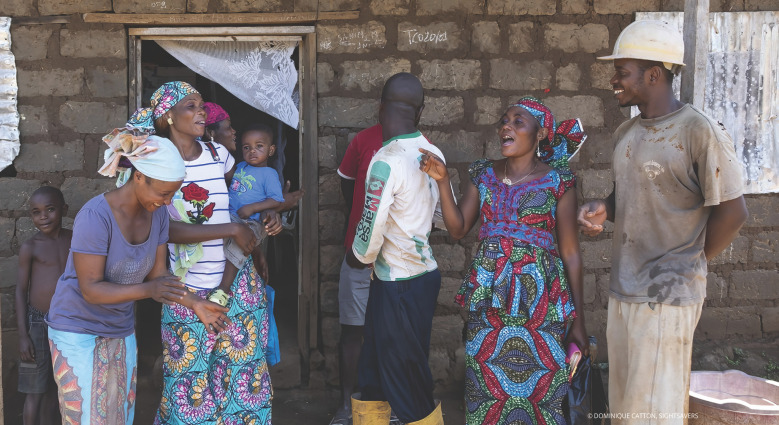
The community plays an important role in the eye health of individuals. cameroon

Community engagement and participation in the health system are fundamental elements of primary health care and are at the heart of the Alma-Ata Declaration of 1978, that: “people have the right and duty to participate individually and collectively in the planning and implementation of their health care.”

At least half of the world's population still does not have full coverage of essential health services, progress towards changing this will only be possible with community participation. A community is a group of people with diverse characteristics linked by social ties, common perspectives, problems, and issues, and who engage in joint action in different or the same geographical locations or settings.

“Empowered people and communities” is one of the three key components of primary eye health.^1^ This means that people know what causes eye disease, what to do to remain healthy, where to go when they become sick, and how to be inclusive of those who have irreversible visual impairment. When communities are engaged and empowered, they can gain the knowledge (health literacy) and ability (agency) to bring about any changes that may be needed.

It is well established that mobilised and empowered communities can play a crucial role in all health processes, such as in planning, allocating resources, delivering services, promoting health, and monitoring health systems. Communities have vast resources – skills, knowledge, and social networks, all of which are building blocks for good eye health. Thus, it is important to enable communities to take control of their health.

Meaningful and effective partnerships between eye health service providers and communities can improve access by bringing services closer to communities, reducing the cost of eye care services, and increasing the efficiency of eye health systems. Community-based eye care activities may be conducted outside the premises of formal health facilities (e.g., hospitals, health centres, and clinics) or in community-based structures (e.g., schools, places of worship, congregate settings, elected local body offices, open groups).

“The responsibility to engage with communities lies with those who have the means.”

By involving local communities in planning and implementing eye health initiatives, communities can become more self-reliant and empowered. Moreover, community engagement can yield positive results, such as improved case-finding and treatment outcomes, raised awareness regarding eye diseases, community advocacy and monitoring of eye care services, and improved uptake of eye health services. Last, but not least, when communities develop strong leadership and the power to assert their health care needs and interests, they have tremendous scope to channel the distribution of resources towards their required health needs. This, in turn, supports eye care providers to deliver effective eye care services that meet real needs.

Community engagement requires that communities and service providers work together, as equal partners. However, the responsibility for initiating, supporting, and sustaining this work lies with those who have the means: mainly service providers, or civil society organisations, who are in a position to support eye care initiatives.

Where possible, service providers should take the initiative to:

- engage with communities and understand their needs
- work with communities to plan eye care services
- provide the eye care services communities need
- be accountable to communities, and give them the means to hold eye care providers accountable.

Understanding the key concepts of community engagement, participation, and accountability is a must for eye care providers. In this issue, we introduce these key concepts and look at examples from different regions. We also discuss how communities can monitor services and provide feedback to improve health care services; and how adopting different intervention strategies ensures that eye care services are inclusive, reach the last mile, are sustainable, and bring positive eye health outcomes.
